# *Streptomyces* sp.—A Treasure Trove of Weapons to Combat Methicillin-Resistant *Staphylococcus aureus* Biofilm Associated with Biomedical Devices

**DOI:** 10.3390/ijms22179360

**Published:** 2021-08-28

**Authors:** Priyia Pusparajah, Vengadesh Letchumanan, Jodi Woan-Fei Law, Nurul-Syakima Ab Mutalib, Yong Sze Ong, Bey-Hing Goh, Loh Teng-Hern Tan, Learn-Han Lee

**Affiliations:** 1 Novel Bacteria and Drug Discovery Research Group (NBDD), Microbes and Bioresource Research Strength (MBRS), Jeffrey Cheah School of Medicine and Health Sciences, Monash University Malaysia, Bandar Sunway 47500, Malaysia; priyia.pusparajah@monash.edu (P.P.); vengadesh.letchumanan1@monash.edu (V.L.); jodi.law1@monash.edu (J.W.-F.L.); syakima@ppukm.ukm.edu.my (N.-S.A.M.); 2 UKM Medical Molecular Biology Institute (UMBI), UKM Medical Centre, Universiti Kebangsaan Malaysia, Kuala Lumpur 56000, Malaysia; 3 Biofunctional Molecule Exploratory Research Group (BMEX), School of Pharmacy, Monash University Malaysia, Bandar Sunway 47500, Malaysia; Ong.YongSze@monash.edu; 4 College of Pharmaceutical Sciences, Zhejiang University, Hangzhou 310058, China; 5 Clinical School Johor Bahru, Jeffrey Cheah School of Medicine and Health Sciences, Monash University Malaysia, Johor Bahru 80100, Malaysia

**Keywords:** methicillin-resistant, *Staphylococcus aureus*, *Streptomyces*, antibiofilm, medical device, anti-MRSA, biofilm-associated infection

## Abstract

Biofilms formed by methicillin-resistant *S. aureus* (MRSA) are among the most frequent causes of biomedical device-related infection, which are difficult to treat and are often persistent and recurrent. Thus, new and effective antibiofilm agents are urgently needed. In this article, we review the most relevant literature of the recent years reporting on promising anti-MRSA biofilm agents derived from the genus *Streptomyces* bacteria, and discuss the potential contribution of these newly reported antibiofilm compounds to the current strategies in preventing biofilm formation and eradicating pre-existing biofilms of the clinically important pathogen MRSA. Many efforts are evidenced to address biofilm-related infections, and some novel strategies have been developed and demonstrated encouraging results in preclinical studies. Nevertheless, more in vivo studies with appropriate biofilm models and well-designed multicenter clinical trials are needed to assess the prospects of these strategies.

## 1. Introduction

Methicillin-resistant *Staphylococcus aureus* (MRSA) is a harmful human pathogen responsible for severe morbidity and mortality worldwide [1]. Emerging multidrug-resistant strains of *S. aureus* pose a significant clinical challenge due to failure in conventional antibiotic therapy [2,3]. Biofilm formation is one of the important pathogen virulent factors that aggravate the increasing spread of antibiotic resistance [4]. Biofilms refer to a cluster of bacterial cells that are enclosed within an extracellular matrix, which collectively attach to an animate or inanimate surface. The cells present within a biofilm pose a major challenge, as they demonstrate increased resistance to antibiotics [5].

Biofilms pose a severe health concern due to the ongoing use of antibiotics, resulting in increased cases of antimicrobial-resistant bacteria that potentially inflict severe infections in patients with indwelling inert surfaces, such as catheters or implants [6]. Biofilms formed by *S. aureus* are among the most frequent causes of biomedical device-related infections that are difficult to treat and are often persistent and recurrent. For instance, the lumen of the central venous catheter is frequently colonized by MRSA embedded in a biofilm layer, causing catheter-related bloodstream infections [7]. The most alarming aspect of biofilm-related infections is that these biofilm-associated cells are highly tolerant to conventional antibiotics, with a 10 to 1000-fold increase in resistance as compared to their planktonic counterpart [8]. Hence, targeting biofilm has become an alternative strategy to control persistent infections associated with MRSA biofilm.

Given the difficulty in eradicating biofilm infections and the limited choices of effective antibiofilm agents in the clinic, efforts are underway to search for and develop approaches to disrupt biofilms, rendering them susceptible to treatment. To search for effective antibiofilm agents, natural products continue to be an important source of drug leads [9,10]. Over the last four decades, more than half of approved drugs under the small molecules category were natural products or directly derived from natural products, while only 33% of the approved drugs were totally synthesized without any natural inspiration [11]. Furthermore, 90 of the 126 (71%) approved small molecules antibacterial drugs were naturally derived or inspired between the year 1981 and 2019 [11]. Interestingly, a vast majority of these antibacterial agents are produced or derived from a group of filamentous Gram-positive soil-dwelling bacteria, under the genus *Streptomyces* [12,13,14]. Besides being important producers of antibiotics, the *Streptomyces* bacteria are well-known for producing bioactive secondary metabolites with diverse pharmacological activities [15,16,17,18,19], including antibiofilm activity.

To improve the current strategies of biofilm inhibition, the aim of the present review is to highlight that the microbial metabolites, specifically from the genus *Streptomyces*, are treasure troves for anti-MRSA biofilm agents, which have the potential for the development of effective and safe therapeutic strategies for MRSA biofilm-associated infections. These anti-MRSA biofilm agents can interfere with different stages of the biofilm developmental process, including to inhibit biofilm formation, mainly via interfering with bacterial adhesion, or disrupt preformed biofilms via destroying the matrix architecture or modulating the complex regulatory systems involved in biofilm formation.

## 2. MRSA and Biofilm Formation on Biomedical Devices

*S. aureus* is a Gram-positive commensal that commonly colonizes the skin and mucosae of a third of the human population, while the remaining people are transiently colonized [20]. The common sites of colonization include the nostrils, skin, axillae, perineum and pharynx [21,22]. Although *S. aureus* appears as a harmless commensal in many people, it is an opportunistic pathogen that is responsible for a wide variety of illnesses, ranging from the common staph infection as boils in adults and impetigo in children, to more severe clinical manifestations such as endocarditis, toxic shock syndrome and indwelling medical devices infections [23].

Being one of the major pathogens for nosocomial infections, *S. aureus* infections in intensive care units are of great health concern, where many reported strains possess a battery of resistance mechanisms against antibiotics. Today, MRSA is highly prevalent in hospital settings and has been the leading cause of morbidity and mortality due to hospital-acquired infection. To further aggravate the situation, *S. aureus* can live in the biofilm state where it is encapsulated in a self-produced extracellular polymeric matrix and adheres to a wide variety of biotic and abiotic surfaces [24,25]. Biofilm formation is a vital virulence factor for the genus *Staphylococcus*, associated with prolonged and recurrent infections of indwelling medical devices [26]. MRSA biofilms are formed on all kinds of catheters, contact lenses, mechanical heart valves and prostheses [27].

Essentially, biofilms provide protection for the bacteria from the antibiotics and host immune system, rendering the bacterial cells within the biofilms more resistant against standard antibiotics, compared to their planktonic counterpart [28]. Furthermore, the bacterial cells exhibit unique phenotypic characteristics within the biofilm matrix, conferring an increased resistance towards common antimicrobial agents. These bacterial cells, known as persister cells, exist as a minor subpopulation at a non-growing state within the biofilm, hence limiting the efficacy of antimicrobial agents that target active cell processes. For instance, a persistent MRSA strain is found to be less susceptible to vancomycin, which is often recommended as first-line therapy for severe MRSA infections [29].

Principally, staphylococcal biofilm formation involves three major life cycle stages: (i) attachment, (ii) maturation and (iii) detachment, to which the expressions of proteinaceous and non-proteinaceous factors are involved in these processes are tightly regulated and species-specific [27]. For example, *S. aureus* possesses various mechanisms to facilitate its adherence to host tissues via surface proteins or foreign material surfaces via hydrophobic interaction. Attachment to an abiotic surface is usually dependent on the physicochemical characteristics of the device and bacterial surfaces; thus, this type of attachment is facilitated by hydrophobic or electrostatic interactions [30,31]. Meanwhile, much more specific interactions are involved in the attachment to biotic surfaces, such as human tissue, where indwelling medical devices are usually covered by host matrix proteins after insertion. Microbial surface components recognizing adhesive matrix molecules (MSCRAMM) protein family is a common group of surface proteins that bind to human extracellular matrix, plasma proteins or directly to host cells [32]. There are a number of MSCRAMM that have been identified and well-studied on *S. aureus*, which include the fibronectin-binding proteins (FnBPA and FnBPB), the fibrinogen-binding clumping factors ClfA and ClfB, serine-aspartate repeat protein family (Sdr) and the collagen binding protein (Cna) [27], where studies also indicated their contribution in promoting *S. aureus* to both indwelling prosthetic devices and plasma-coated biological surfaces [33,34,35].

After the initial attachment, the bacteria progress into a growth and maturation phase, where the bacteria proliferate and form multi-layered microcolonies. During the proliferation phase, these bacteria secrete and eventually embed themselves within the matrices of extracellular polymeric substance (EPS) that are primarily composed of extracellular polysaccharide intercellular adhesion (PIA), *S. aureus* surface protein G (SasG), teichoic acids, accumulation-associated protein (Aap) and extracellular DNA (eDNA) [36]. PIA, also termed poly-N-acetyl-glucosamine (PNAG), is a crucial adhesive biofilm polysaccharide molecule that is synthesized, exported and modified by the products of *ica* gene locus (*icaA*, *icaD*, *icaB* and *icaC*). Thus, the composition of the biofilm matrix for both *ica*-positive and *ica*-locus negative strains of *S. aureus* is extremely different, whereby PIA is the main component of the *ica*-locus positive strain *S. aureus* while the main component of the *ica*-locus negative strain *S. aureus* is mainly made of eDNA [37]. PIA is indispensable for staphylococcal biofilm formation, providing positively charged surfaces for adherence of bacterial cells to PIA polymer [38]. Meanwhile, other specific proteins, such as Aap and SasG, are shown to be important for adherence to host cells and interbacterial aggregations, particularly for those staphylococcal strains that are devoid of *ica* genes and do not produce PIA [39,40].

In the third stage of biofilm development, biofilm disassembly, which converts bacterial cells in the biofilm to their planktonic state, is an important process that drives medical device-associated infections. This is because the detached bacterial cells from the surface of an indwelling medical device may re-establish local infection, or travel to distant sites of the human body and cause bloodstream infection [38]. The detachment is facilitated by the expression of surfactant-like peptides, such as proteases, toxins and phenol-soluble modulins (PSMs), to which they are regulated by the accessory gene regulator (*agr*) system that helps in the breaking down of the biofilm matrix [41].

## 3. *Streptomyces* sp. a Valuable Source for Anti-MRSA Biofilm Agents

*Streptomyces* are Gram-positive, filamentous, spore-forming bacteria with high G + C content under the phylum Actinobacteria [42]. *Streptomyces* bacteria are ubiquitously found in the soils from various environments as free-living organisms and symbionts of plants and animals [43]. Within the phylum Actinobacteria, *Streptomyces* stand out as the most prolific producer of commercially and therapeutically important bioactive compounds, where approximately 50% of clinically relevant antibiotics are produced or derived from the secondary metabolites of *Streptomyces* bacteria [1,44].

The prominent role of *Streptomyces* in antibacterial drug discovery began with the isolation of actinomycin and streptomycin by Waksman and Woodruff, who developed the ‘Waksman platform’, being the most successful antibacterial drug discovery platform that applies a systematic screen of soil bacteria during the golden era of antibiotic discovery [45]. The platform has paved the way to the main antibacterial classes and the discovery of clinically important antibiotics available today, including tetracycline, chloramphenicol, erythromycin, vancomycin, rifamycin, daptomycin and gentamicin. Although the Waksman platform began to wane in the 1960s, due to the rediscovery of known antibiotics together with the rise and spread of resistant pathogens [46], many efforts have been invested in developing a novel and sustainable antibiotic discovery platform for the past decades [47]. In recent years, the uprising of various next-generation sequencing (NGS) platforms has revolutionized the field of natural product discovery, where the rapidly expanding microbial genomic and metagenomic datasets unravel a vast number of biosynthetic gene clusters that encode a lot more natural products than what has been characterized to date [48]. For instance, recent advances in genome mining have revealed that *Streptomyces* genomes contain numerous previously unknown cryptic biosynthetic clusters, suggesting that *Streptomyces* genomes remain a valuable resource for novel drug discovery [49,50]. Given that the unparallel contribution of *Streptomyces* bacteria to human health is a treasure trove of antibacterial agents [51], there is an increasing trend of studies that show renewed interest in the potential of *Streptomyces* bacteria in the production of antibiofilm compounds.

Based on the literature, there are numerous reports on the isolation of *Streptomyces* sp. from different sources that possess the ability to produce secondary metabolites with anti-MRSA biofilm activities [52]. These studies performed a systematic screen of antibiofilm activities with the crude extract of *Streptomyces* sp. fermentation supernatant, followed by chemical analysis to identify the presence of potential antibiofilm agents. For example, Singh and Dubey [53] recently reported that a novel endophytic strain, *Streptomyces californicus* strain ADR1 isolated from a medicinally important plant *Datura metel*, has the ability to produce secondary metabolites with promising anti-MRSA biofilm activity. The study showed that the ethyl acetate crude extract of supernatant inhibited biofilm formation and preformed biofilm of MRSA with a 90% biofilm inhibitory concentration (BIC_90_) at 4.59 μg/mL and 19.64 μg/mL, respectively [53].

In recent years, there has been an increased interest in the underexplored marine *Streptomyces* as a source of bioactive substances [54], including antibiofilm agents. Marine *Streptomyces* are widely distributed in biological sources, such as fishes, molluscs, sponges, seaweeds, mangroves and deep-sea sediments [55,56,57]. For instance, Bakkiyaraj and Karutha Pandian [58] isolated a marine actinomycete, *S. akiyoshiensis* CAA-3, from a coral reef ecosystem, to which the crude extract of CAA-3 showed 75–80% biofilm inhibition against MRSA at 100 μg/mL in vitro. The study also suggested a low risk of the pathogens developing resistance towards the crude extract CAA-3, given that it does not interfere with the growth of the pathogen [58]. Similarly, a few more studies demonstrated the isolation of *Streptomyces* sp. from the marine ecosystems, such as sponges, to exhibit antibiofilm potentials [59,60,61]. The crude extract of *Streptomyces* sp. SBT348, which was isolated from marine sponge *Petrosia ficiformis*, was demonstrated to contain putatively identified compounds with promising anti-MRSA biofilm activities, such as azlomycin, streptocytosine B, streptocytosin C, daryamide A, azamerone, antimycin B1, usabamycin A and actinoramide D [60]. A follow-up study revealed the isolation of a potentially novel compound, SKC3 from the SBT343 extract, and suggested that it exerts anti-staphylococcal biofilm activity via the downregulation of purine biosynthetic genes [59]. Meanwhile, another study showed a marine *Streptomyces griseoincarnatus* HK12, isolated from marine sponge *Callyspongia* sp., produces secondary metabolites, such as 13Z-octadecenal, 9Z-octadecenal, arachidic acid, tetracosanoic acid and erucic acid, with anti-staphylococcal biofilm activity. In addition to this, a study reported that the supernatant of *Streptomyces* sp. BFI 250 contains extracellular proteases, which can significantly reduce biofilm formation and enhance biofilm dispersal of *S. aureus* [62].

### Newly Reported Anti-MRSA Biofilm Compounds Synthesized by Streptomyces Bacteria

**Figure 1:**
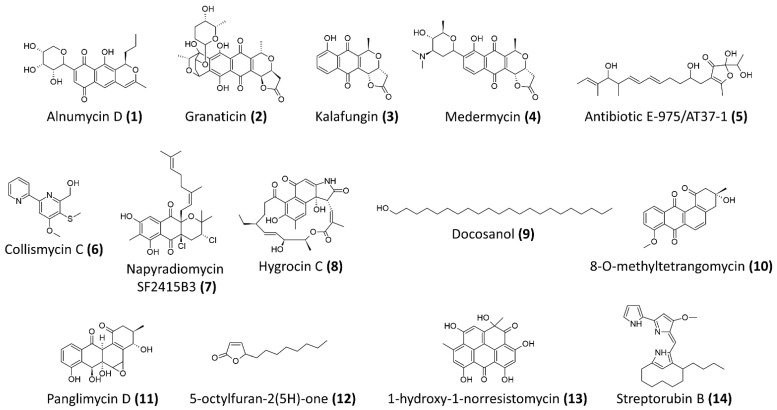
The chemical structures of antibiofilm compounds derived from *Streptomyces* sp.

In recent years, there have been various bioactive compounds with anti-staphylococcal and anti-MRSA biofilms activity in a wide variety of chemotypes, reported from drug discovery studies investigating the biosynthetic potentials of *Streptomyces* bacteria. Several bioactive compounds, isolated from *Streptomyces* sp., were shown to exhibit promising antibiofilm activities that prevent biofilm formation and disrupt the preformed biofilms of *S. aureus* and MRSA at the micromolar range, including alnumycin D (**1**), granaticin B (**2**), kalafungin (**3**), medermycin (**4**) [63], AT37-1 (**5**) [64], collismycin C (**6**), napyradiomycin SF2415B3 (**7**) [65], hygrocin C (**8**) [66], 8-O-metyltetrangomycin (**10**) [67], antibiotic 5812-A/C [68], panglimycin D (**11**) [69] and streptorubin B (**14**) [70] (Table 1). The chemical structures of these compounds are illustrated in Figure 1.

Alnumycins are pyranonaphthoquinone polyketides recently isolated from *Streptomyces* bacteria that exhibit antibiofilm activities against *S. aureus.* Among the alnumycins isolated from the different strains of *Streptomyces albus*, alnumycin D (**1**) exhibited 100% killing of *S. aureus* biofilm cells at 40μM [63]. Interestingly, alnumycin D (**1**) demonstrated strong efficacy in inhibiting preformed biofilms and planktonic growth with IC_50_ measured at 2.66 μM and 1.75 μM, respectively. Similar to another pyranonaphthoquinone polyketides, granaticin B (**2**), which was isolated from *Streptomyces violaceoruber* Tü22 in the study, alnumycin D (**1**) was shown to be as highly active as granaticin B (**2**), and both were equally efficacious against biofilm cells and planktonic cells [63]. A further comparison was performed, in terms of the antibiofilm activity between several other pyranonaphthoquinone polyketides, such as kalafungin (**3**) and medermycin (**4**) (all was isolated *Streptomyces* bacteria), and suggested that the oxygenation pattern of the lateral naphthoquinone ring on these compounds has a significant impact on the antibiofilm potency [63].

An anti-staphylococcal biofilm metabolite, named AT37-1 or antibiotic E-975 (**5**), was reported from a *Streptomyces* sp. derived from Saharan soil sample in Adrar. The study indicated that this compound belongs to the group of furanone and the heterocycles family, achieving 50% biofilm inhibitory activity against methicillin-susceptible *S. aureus* (MSSA) and MRSA, at concentrations between 5 to 10 μg/mL [64].

A recent study showed the isolation of collismycin C (**6**) from the fermentation supernatant of a marine-derived *Streptomyces* sp. MC025, isolated from marine organisms in the waters of Micronesia. The study demonstrated that collismycin C (**6**) exhibits potent antibiofilm activity against both MSSA and MRSA [71]. Collismycin C (**6**) at 50 μg/mL successfully reduced the biomass of *S. aureus* 6538 and MRSA biofilms by >98% and 90%, respectively. Furthermore, the study suggested that collismycin C (**6**), as a 2,2′-bipyridine containing compound, exerts its antibiofilm activity against staphylococci by chelating iron ions [71].

Antimicrobial peptides (AMPs) are host defense peptides, exhibiting a broad spectrum of antimicrobial activities. AMPs can destabilize and permeabilize bacterial cell membranes via the interactions between their net positive charge characteristic and the negatively charged bacterial membranes or other cell components, such as lipopolysaccharides [75]. Vasilchenko et al. [68] reported the isolation of presumably a cyclic (lipo)glycopeptide, or antimicrobial peptide complex 5812-A/C, which confers a potent antimicrobial effect against planktonic cells of Gram-positive pathogens, including MRSA and MSSA with MIC of 3.1 μg/mL and 0.8 μg/mL, respectively. The study suggested that AMP complex 5812-A/C exhibits calcium-dependent bactericidal activity. On top of this, AMP complex 5812-A/C can eradicate mature *S. aureus* biofilm, whereby it elicits antibiofilm activity by penetrating and inhibiting the metabolic activity of bacterial cells within the mature biofilm [68].

## 4. Clinically Used Antibiotics Derived from *Streptomyces* Bacteria for Medical Device-Related MRSA Biofilm Infections

Clinical studies have shown a strong correlation between the use of foreign medical bodies or indwelling devices and biofilm infection [76]. The current clinical approaches for the treatment of biofilm-associated infections are principally comprised of the removal of colonized foreign bodies, followed by the replacement with new ones, surgical debridement, drainage of abscesses and aggressive antibiotic therapy [4]. Nevertheless, early treatment is the best strategy for the management of biofilm-related infection. However, early detection for biofilm-related infection is often difficult, and the development of antibiotic resistance by *S. aureus* further poses a significant challenge to antibiotic therapy.

Current clinical guidelines recommend the administration of prophylactic antibiotics, such as cefuroxime and cefazolin, an hour prior to implantation surgery against Gram-positive staphylococci, while vancomycin and clindamycin are often given as an alternative to patients with β-lactam allergy or MRSA infection [77]. For the treatment of implant and catheter-related infections, parenteral vancomycin or daptomycin is the cornerstone of an empirical regime in settings with a high prevalence of MRSA [78,79]. However, many of these antibiotics were shown to be ineffective for the treatment of MRSA infection, involving biofilm in vivo [80,81]. In addition, rifampin has been recommended as an agent in combination therapy in orthopaedic device-related infections [82], whereby it has been reported to have some activity against staphylococcal biofilms, but is not recommended as a monotherapy due to the rapid selection of resistant mutants [83].

Achieving total eradication of the biofilm-associated infections with these approaches is challenging, and is often dependent upon causative agents and the infection site. Moreover, a long-term administration of antibiotics to suppress the biofilm from growing is required if removal is not possible [76]. Another concern with the use of antibiotics is the fact that several studies have indicated that several commonly prescribed antibiotics at a sub-inhibitory concentration may induce biofilm formation [84]. Sub-inhibitory antibiotic concentration often occurs at the beginning and end of a dosing regimen, and most importantly, it also occurs within the biofilm where antibiotics are not readily penetrable into the biofilm matrix [85]. For example, vancomycin is a glycopeptide antibiotic, extensively used as first-line therapy for severe MRSA infection, yet a high rate of vancomycin treatment failure in vancomycin-susceptible MRSA infections has been observed [80,81]. The inefficiency of vancomycin in treating MRSA biofilm-associated infection has been associated with its reduced biofilm penetrating ability [86,87].

Nevertheless, many efforts have been devoted to investigating the effectiveness of different clinically used antibiotics to treat biofilm-associated staphylococcal infections, especially from the *Streptomyces*-derived antibiotics or its derivates, which include vancomycin, fosfomycin, rifampicin, clindamycin, daptomycin, azithromycin [88], tigecycline and minocycline [89,90]. A study demonstrated that antibiotics daptomycin, tigecycline and rifampin are effective to be used as antimicrobial lock therapy to treat device-related staphylococcal infections involving biofilms [91]. Interestingly, daptomycin, tigecycline and rifampin conferred a more sustained antibiofilm activity against mature biofilms (3 to 5 days old) as compared to vancomycin, which failed to eradicate older biofilms, in a novel in vivo-relevant model of catheter-related infection mediated by MRSA [91]. Another study demonstrated that minocycline is effective in inhibiting biofilm formation and eradicating mature biofilms in both *ica*-positive and *ica*-locus negative MRSA, in comparison to vancomycin [92], indicating that the efficacy of different antibiotics is dependent on the different biochemical composition of MRSA biofilm.

The synergistic antibacterial activity between these antibiotics has been evaluated against *in vitro* and in vivo MRSA biofilm models [93,94]. Based on the theory of the mutant selection window, it is supposed that the combined use of antibiotics with a different mechanism of action may yield good outcomes in biofilm eradication [95]. The combination therapy not only can prevent mutant selection due to the different bactericidal mechanisms exhibited by multiple antibiotics, but can also increase the efficacy and reduce the side-effects of the antibiotics [95]. For instance, Shi et al. [94] demonstrated the synergistic bactericidal effect of combining vancomycin and fosfomycin in treating chronic biofilm-associated MRSA infection in a rat carboxymethylcellulose-pouch model. In comparison to mono-administration, the study showed that combination therapy confers better activity in eradicating mature biofilm via modifying the structural component of the biofilm as well as ameliorating the inflammatory response associated with the biofilm [94].

The repurposing of drugs has become an attractive strategy in the search for medicine without going through the exhaustive process of de novo pharmacological optimization. Given that the safety and pharmacokinetic profiles are known for the approved or investigational drugs, drug repurposing can reduce the risk of failure, time and costs within the drug development pipeline, while a traditional de novo drug discovery and development pipeline typically can be a 10- to 17-year pathway to the market [96]. There are several potential antibiofilm activities that have been evidenced in drugs that were originally developed for treating noninfectious human diseases, such as antineoplastic drugs. For instance, dactinomycin or actinomycin D is an antitumor drug that was approved by the US Food and Drug Administration (FDA) in 1964 for treating many tumours, including Wilms’ tumour, childhood rhabdomyosarcoma, Ewing’s sarcoma and non-seminomatous testicular cancer [97]. Interestingly, a study recently reported that actinomycin D exhibits anti-biofilm activity against multiples strains of *S. aureus*, including *S. aureus* MSSA 25923, *S. aureus* MSSA 6538 and *S. aureus* MRSA 33,591 [98]. The antibiofilm activities of dactinomycin against *S. aureus* were mediated by reducing slime production, α-toxin production and cell surface hydrophobicity [98]. In addition, the study successfully fabricated a biodegradable poly(lactic-co-glycolic acid) (PLGA) film incorporated with actinomycin D that prevents MRSA biofilm formation, suggesting the potential development of PLGA/actinomycin D as an antibiofilm coating on medical devices [98].

Another interesting example is ivermectin, which is an FDA-approved antihelminthic drug that has been shown for the first time to possess antibacterial activity against clinical isolates of *S. aureus*, which include both MSSA and MRSA with a MIC value of 6.25 μg/mL and 12.5 μg/mL, respectively [99]. A number of studies also evaluated its antibiofilm activity against MRSA but did not yield promising results [100,101]. Remarkably, substituting the 4″-positive hydroxyl group with an amino group resulted in a novel ivermectin-derived compound (D4) that demonstrated an improved antibiofilm activity against MRSA compared to its parent compound. The study further elucidated that D4 may exert anti-MRSA biofilm by downregulating several biofilm formation-related genes, such as *spA* and *icaD*, which encode proteins involved in the attachment and biofilm formation of *S. aureus* [100].

## 5. Targeting MRSA Biofilm in the Treatment of Biomedical Device-Related Infections

**Figure 2:**
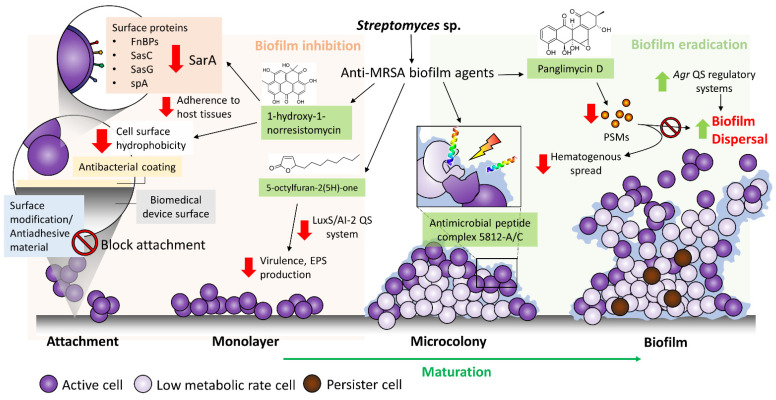
The strategies of biofilm inhibition and biofilm eradication employed by the anti-MRSA biofilm agents derived from *Streptomyces* sp.

The identification and development of compounds that target MRSA biofilms are as important as the development of new antibiotics in the treatment of staph infections. These anti-MRSA biofilm strategies can be broadly divided into two categories, which include (1) inhibition of biofilm formation (typically these anti-biofilm compounds ideally exhibit no or little toxicity toward planktonic bacteria) and (2) dispersion or eradication of preformed biofilms (Figure 2). In terms of their therapeutic applications, the former strategy is useful to prevent biofilm formation after surgery or on medical devices, whereas the latter strategy is suitable to combine the anti-MRSA biofilm agents with standard drugs to target the biofilm-forming MRSA within the infection sites.

Given that attachment of bacterial cells to the surface or substratum is the first step of successful biofilm formation, preventing the initial colonization of MRSA on surfaces of medical devices is effective to reduce biofilm-related infection. These approaches may include the application of antibiofilm coatings that interfere with the attachment of bacterial cells to the device surfaces, or surface modification of the biomaterials that prevent bacterial attachment. Primarily, the incorporation of chemicals or the development of materials aims to modify the surface’s physical properties, including the hydrophobicity and hydrophilicity, surface roughness and texture of the surface, such that bacteria are no longer able to attach easily. Over the years, there have been various types of bacteriostatic and bactericidal coatings developed for preventing the attachment of bacteria, including *S. aureus*, on biomedical devices [102,103,104,105]. A study, which is worthy of being mentioned here, by Lee et al. [98] successfully fabricated biodegradable PLGA films containing daptomycin, that markedly reduced MRSA biofilm formation on glass surface. The antibiofilm efficacy of PLGA/daptomycin coating on glass against MRSA was suggested to be mediated by reducing the cell surface hydrophobicity and thus inhibiting the attachment of the bacterial cells [98]. Nonetheless, there are several important considerations that should be taken while developing antiadhesive surfaces or antibacterial coatings, which include preserving the primary function of the biomedical devices and ensuring the materials or coatings are not toxic to host cells [106,107].

Alternatively, targeting the surface proteins of MRSA with chemical compounds to disrupt the adhesion or adherence process to host cells could be an attractive approach to mitigate biofilm formation. However, a much clearer understanding of how the bacteria coordinate the expression of the surface effectors and how the various surfaces interact with these effectors is needed for one to develop effective strategies that are primarily focusing on the initial attachment of the bacterial cells to surfaces. To date, surface proteins that have been known to play a specific role in biofilm formation include ClfB, FnBPs, surface proteins SasC, SasG and protein A (spA) [108]. Ramalingam et al. [74] reported that a *Streptomyces*-derived anti-MRSA biofilm compound, 1-hydroxy-1-norresistomycin (**13**)**,** interacts with the staphylococcal accessory regulatory (SarA) protein of *S. aureus* via a molecular docking analysis, suggesting that the anti-MRSA biofilm activity of compound (**13**) may be mediated by suppressing the expression of important surface proteins of *S. aureus*. SarA is a DNA binding regulatory protein that directs the expression of various virulence genes in *S. aureus*. For instance, SarA binds to the upstream promoter regions of several target genes of FnbA and FnbB, spA and PIA synthesis proteins [109,110]. In addition to this, 1-hydroxy-1-norresistomycin (**13**) was shown to reduce the cell surface hydrophobicity of *S. aureus*. The cell surface hydrophobicity is said to be important in facilitating the attachment of the bacteria to surfaces of biomedical devices, which are predominantly hydrophobic in nature. Thus, the reduction of cell surface hydrophobicity in *S. aureus* upon exposure to 1-hydro-1-norresistomycin (**13**) may render the bacterial cells ineffective in surface attachment, hence limiting biofilm formation [74].

Disruption of mature biofilms is an attractive approach to reduce the protective effects of the biofilm matrix conferred for the bacterial cells embedded within it. Given the EPS matrix is mainly composed of polysaccharides, structural proteins and extracellular DNA, targeting the EPS matrix with specific matrix-degrading molecules could be a promising approach to eradicate the biofilms by destroying the protective matrix and rendering the protected bacteria cell sensitive to other treatments. There are several well-known enzymes, such as dispersin B and thermonuclease/DNase, that can be applied exogenously to disrupt the polysaccharide and eDNA components of EPS matrix, respectively [111]. *Streptomyces* sp. bacteria have been known as prolific producers of extracellular enzymes, including proteases and DNases, that can potentially be developed into anti-MRSA biofilm agents [62,112].

With a better understanding of the complex regulatory systems of the biofilm dispersal event of *S. aureus*, *S. aureus*-specific factors that initiate biofilm disassembly represent attractive targets for developing a strategy to manage biofilm-associated infection by manipulating the natural staphylococcal disassembly mechanisms. For example, PSMs are surfactant-like peptides that promote biofilm disassembly [113], while the soluble PSM peptides form insoluble amyloid-like fibers during biofilm formation [114]. Queck et al. [115] demonstrated that formylated phenol-soluble modulin (PSM-mec) isolated from hospital-acquired MRSA plays a role in promoting biofilm formation on surfaces of medical devices. The key role of PSMs in biofilm structuring and detachment of *S. aureus* is further proven by the impaired biofilm maturation and dissociation in *S. aureus psm* deletion mutants [113]. Recently, an aromatic polyketide, panglimycin D (**11**), derived from *Streptomyces* sp., was reported to significantly inhibit the production of PSMs by MRSA [69], suggesting that it could be developed as a potential preventive agent for biofilm-associated infection in catheterized patients. In a previous study, inactivation of PSMs by antibodies was shown to prevent the hematogenous spread of *S. epidermidis* in an in vivo catheter model [116].

Inhibition of quorum sensing (QS), which also refers to bacterial cell-cell communication, is another promising strategy to limit biofilm formation [117]. QS is a process that involves the bacterial communication system at a molecular level regulating the expression of genes involved in virulence, adhesion and biofilm formation. Generally, Gram-negative bacteria secrete acyl-homoserine lactones (AHLs) as the small diffusible signaling molecules, called autoinducers (AIs), that are mediated by a LuxI/LuxR-type system, while QS in Gram-positive bacteria, oligopeptide, AIs are produced and regulated by two-component systems [118]. In addition, a QS system that involves the production of auto-inducer 2 (AI-2) is shown to be shared by both Gram-positive and Gram-negative bacteria [119]. In *S. aureus*, the *agr* system is the major quorum-sensing system and appears to be distinct from other bacterial systems in the regulation of biofilm development, whereby *agr* is repressed in biofilms. Conversely, activation of the *agr* QS network via the release of the auto-inducing peptide (AIP) results in dispersal of *S. aureus* biofilm. Therefore, developing agents that activate *agr* QS system could be a useful strategy to eradicate *S. aureus* from mature biofilm. Meanwhile, agents that inhibit *agr* QS system potentially block the production of virulence determinants that are important at the beginning of an infection, such as by reducing the production of surface components required for initial colonization of tissues [120]. Besides *agr*, the role of LuxS, an enzyme (*S*-ribosylhomocysteine lyase) involved in AI-2 synthesis in staphylococcal quorum-sensing, which is less understood, has been associated with capsule synthesis, biofilm formation and virulence via AI-2 regulation [121,122]. A more recent study demonstrated that the LuxS/AI-2 system could regulate PIA-dependent biofilm formation by suppressing the expression of *rbf*, which is a positive regulator of biofilm formation in *S. aureus* [119]. Although the QS regulatory systems in *S. aureus* await further elucidation, targeting the LuxS/AI-2 system can be a successful anti-infective strategy for *S. aureus* biofilms. For instance, Yin et al. [73] demonstrated the anti-MRSA biofilm activity of 5-octylfuran-2(*5H*)-one (**12**), a butanolide derived from *Streptomyces* sp., could be mediated via non-specific quorum-sensing inhibitions, reducing both AI-2 and AHLs; however, more study is required to elucidate the exact antibiofilm mechanism. Nonetheless, several previous investigations revealed the anti-staphylococcal biofilm mechanism of 2(*5H*)-furanone derivatives mediated via the inhibition of LuxS QS system [123,124]. A study by Zang et al. [125] even showed that brominated furanone targets LuxS induce covalent modification, and thus inactivation of LuxS.

## 6. Conclusions and Future Outlook

Infections involving multidrug-resistant pathogens, including MRSA, have been the most challenging issues haunting the healthcare system for the past decade. The ability to form biofilms that protect the bacterial cells from host defenses and antibiotic therapy has contributed to the majority of chronic staphylococcal infections, especially those associated with indwelling medical devices. Conventional antibiotic therapy alone often fails to eradicate MRSA biofilm. Up until today, there is no approved drug that acts specifically against MRSA biofilms in clinical trials, indicating there is an urgent need for alternative strategies. Therefore, significant efforts should be made to identify more efficient therapeutic approaches targeting MRSA biofilms, with a deeper understanding of their mode of action and in vivo efficacy.

*Streptomyces* bacteria have been demonstrated in numerous studies as valuable sources for antibiotics and natural anti-biofilm agents that could be added to the arsenal in the treatment of medical device-associated MRSA infections. Given that most monotherapies show poor efficacy in treating MRSA biofilm-associated infection, combining multiple antibiotics or antimicrobials has shown some promising data in the management of biofilm-associated infections. The idea of repurposing previously used drugs should be encouraged further, especially to reduce the prolonged and disconcerting process of discovering new drugs. For instance, dactinomycin and ivermectin are two promising candidates for future treatment of biofilm-associated infection. Although many studies have shown encouraging results from the anti-MRSA biofilm compounds isolated from *Streptomyces* sp. in recent years, many gaps in knowledge are yet to be uncovered due to the lack of in vivo studies with appropriate biofilm models and elucidation of the mechanisms of action.

The growing knowledge of staphylococcal biofilm genetics has improved our understanding of the various phases of the biofilm cycle, and helped us in better devising potential therapeutic strategies for medical device-associated infection. The advances in nanotechnologies and surface materials have led to the fabrication of surfaces and coatings with antibacterial agents, presenting as attractive strategies to prevent the initial attachment and colonization of the pathogens [126,127]. With a better knowledge of the biofilm matrix and dispersal mechanism of biofilm, several promising biofilm-targeting strategies have been developed, such as the use of EPS matrix-degrading enzymes and small molecules that modulate regulatory systems that are involved in biofilm formation and dispersal. To ensure the clinical relevance of these antibiofilm agents, more rigorous studies are required for their drug-like property, effective dosage and proper route of delivery to a specific site in an in vivo system. Moreover, with the advancement of molecular biology and bioinformatics and the valuing of microbial resources, more research could focus on genome mining methods to effectively improve the success rate of discovering novel antibiofilm compounds of *Streptomyces* bacteria. This would accelerate the development of antibiofilm compounds in clinical studies and would be a crucial step forward in the combat against antimicrobial resistance.

## Figures and Tables

**Table 1:** Potential anti-staphylococcal/MRSA biofilm agents derived from *Streptomyces* bacteria.

Compound Name	Chemical Class	*Streptomyces* Producer and Isolation Source	Anti-Staphylococcal/MRSA Biofilm Activity	Reference
Alnumycin D (**1**)	polyketides	*Streptomyces albus* (pAlnuoriΔaln6)	Resazurin-based viability assay Preexposure IC_50_ against planktonic cells of *S. aureus* ATCC 25,923 = 2.66 μM Preexpsoure IC_50_ against biofilm cells of *S. aureus* ATCC 25,923 = 1.75 μM Postexposure IC_50_ against preformed ATCC 25,923 biofilms = 4.02 μM	[63]
Granaticin B (**2**)	polyketides	*Streptomyces violaceoruber* Tü22	Resazurin-based viability assay Preexposure IC_50_ against planktonic cells of *S. aureus* ATCC 25,923 = 2.61 μM Preexpsoure IC_50_ against biofilm cells of *S. aureus* ATCC 25,923 = 2.76 μM Postexposure IC_50_ against preformed ATCC 25,923 biofilms = 3.72 μM	[63]
Kalafungin (**3**)	polyketides	*Streptomyces tanashiensis* Kala	Resazurin-based viability assay Preexposure IC_50_ against planktonic cells of *S. aureus* ATCC 25,923 = 1.11 μM Preexpsoure IC_50_ against biofilm cells of *S. aureus* ATCC 25,923 = 3.87 μM Postexposure IC_50_ against preformed ATCC 25,923 biofilms = 27.8 μM	[63]
Medermycin (**4**)	polyketides	*Streptomyces coelicolor* CH999/pIK340	Resazurin-based viability assay Preexposure IC_50_ against planktonic cells of *S. aureus* ATCC 25,923 = 2.81 μM Preexpsoure IC_50_ against biofilm cells of *S. aureus* ATCC 25,923 = 2.5 μM Postexposure IC_50_ against preformed ATCC 25,923 biofilms = 24.6 μM	[63]
Antibiotic E-975 (**5**)	Heterocyclic furanone	*Streptomyces* sp. AT37	Minimum concentration for 50% inhibition of biofilm formation of *S. aureus* ATCC 25,923 and MRSA ATCC 43,300 = 15 μg/mL and 10 μg/mL, respectively	[64]
Collismycin C (**6**)	Polyketides-nonribosomal peptides	*Streptomyces* sp. MC025	Significant inhibition of biofilm formation by MRSA, ATCC 33,591 at concentration >5 μg/mL At 10 μg/mL, more 50% inhibition against biofilm formation and no antibacterial activity against the bacterial growth	[71]
Napyradiomycin SF2415B3 (**7**)	Hybrid isoprenoids	*Streptomyces* sp. MAR4, marine sediments from Madeira Archipelago	Minimum biofilm inhibitory concentration of 15.6 μg/mL—inhibits biofilm formation of *S. aureus* NCTC8325-4	[65]
Hygrocin C (**8**)	Ansamycin, lipopeptides	*Streptomyces* sp. SCSGAA 0027, South China Sea gorgonian *Subergorgia suberosa*	Minimum concentration for 80% inhibition of biofilm formation of *S. aureus* ATCC 6538 = 25 μg/mL	[66]
Docosanol (**9**)	Aliphatic alcohol	*Streptomyces griseus* TBG19NRA1	Around 80% reduction in biofilm formation at concentration >500 μg/mL	[72]
Antibiotic 5812-A/C	Antimicrobial peptide complex	*Streptomyces roseoflavus* INA-Ac-5812	More than 50% reduction of preformed biofilms of *S. aureus* 209P at 1.8 μg/mL Penetrate and inhibit the metabolic activity of *S. aureus* 209P in preformed biofilms	[68]
8-O-metyltetrangomycin (**10**)	Angucycline, aromatic polyketides	*Streptomyces* sp. SBRK-2, marine sponge *Spirostella* sp.	At 2 μg/mL, 70% inhibition of biofilm formation by *S. aureus* ATCC 25923 Membrane damaging and increased cell surface hydrophobicity	[67]
Panglimycin D (**11**)	Angucyclinones, aromatic polyketides	*Streptomyces bulli* GJA1, endophyte of *Gardenia jasminoides*	At 5 μg/mL, biofilm formation of MRSA USA300 was inhibited by 40% Inhibited the production of PSMα2, PSMα3, PSMα4, and δ-toxin of MRSA USA300	[69]
5-octylfuran-2(*5H*)-one (**12**)	Butenolides, furanones	Marine-derived *Streptomyces* sp.	100% inhibition of biofilm formation and eradication of preformed biofilm of MRSA ATCC43300 at 200 μg/mL, while minimum inhibitory concentration of >1200 μg/mL Inhibition of autoinducer-2 and acyl-homoserine lactone, suggested it could be a non-specific quorum-sensing inhibitor	[73]
1-hydroxy-1-norresistomycin (**13**)	Pentacyclic polyketides	*Streptomyces variabilis*, Scleractinia coral *Acropora Formosa*	93% inhibition of biofilm formation by *S. aureus* at 200 μg/mL Reduced *S. aureus* cell surface hydrophobicity Docking study showed good affinity towards SarA and ScpA protein of *S. aureus*	[74]
Streptorubin B (**14**)	Prodiginine, bacterial alkaloids	*Streptomyces* sp. strain MC11024, soil sample from Suita, Osaka, Japan	IC_50_ of biofilm inhibition against MRSA N315 = 0.22 μg/mL (0.56 μM), minimum inhibitory concentration of growth = 32 μg/mL	[70]

## Abbreviations

MRSA	methicillin-resistant *Staphylococcus aureus*
MSCRAMM	microbial surface components recognizing adhesive matrix molecules
FnBP	fibronectin-binding protein
Clf	clumping factor
Sdr	serine-aspartate repeat protein
Cna	collagen binding protein
EPS	extracellular polymeric substance
PIA	polysaccharide intercellular adhesion
SasG	*S. aureus* protein G
Aap	accumulation-associated protein
eDNA	extracellular DNA
PNAG	poly-N-acetyl-glucosamine
PSM	phenol-soluble modulin
agr	accessory gene regulator
NGS	Next-Generation Sequencing
BIC_90_	90% biofilm inhibitory concentration
MSSA	methicillin-susceptible *S. aureus*
AMP	antimicrobial peptide
IC_50_	50% inhibitory concentration
PLGA	poly(lactic-co-glycolic acid)
spA	protein A
SarA	staphylococcal accessory regulatory protein
QS	quorum sensing
AHL	acyl-homoserine lactone
AIs	autoinducers
AI2	autoinducer 2
AIP	auto-inducing peptide
